# Saving Mothers, Giving Life: A Systems Approach to Reducing Maternal and Perinatal Deaths in Uganda and Zambia

**DOI:** 10.9745/GHSP-D-19-00037

**Published:** 2019-03-11

**Authors:** Lois Quam, Angeli Achrekar, Robert Clay

**Affiliations:** a Director, Global Health Initiative, U.S. Department of State. Now with Pathfinder International, Boston, MA, USA.; b Principal Deputy Coordinator (acting), U.S. Department of State. Office of the U.S. Global AIDS Coordinator and Health Diplomacy, Washington, DC, USA.; c Deputy Assistant Administrator, Bureau for Global Health, U.S. Agency for International Development. Now with Save the Children USA, Washington, DC, USA.

## Abstract

The 5-year public-private partnership boldly addressed maternal mortality in Uganda and Zambia using a systems approach at the district level to avoid delays in women seeking, reaching, and receiving timely, quality services. This supplement provides details on the Saving Mothers, Giving Life partnership and approach, including the model, impact, costs, and sustainability.

## WHAT CHALLENGE DID WE FACE?

Despite all the gains of the last 30 years in global health and development, maternal mortality is often regarded as an intractable problem. Complications during pregnancy, childbirth, or in the 42 days after birth were the leading causes of death among women of reproductive age when Saving Mothers, Giving Life was initiated and remain so today Saving Mothers, Giving Life initiative and remain so today.^1^ At the outset of Saving Mothers, nearly 30 women died every hour, 800 women died each day, and an estimated 287,000 women died each year due to pregnancy- and childbirth-related causes.^1^ An additional 15–20 million women suffered debilitating infections and disabilities annually because of pregnancy.^1^ Co-infection with HIV was increasingly one of the most common causes of pregnancy-associated deaths in Africa (ranging from 15% to 40%).^1^ Yet mothers were dying for reasons that were well understood and almost always preventable, even in the poorest countries. Interventions to lower maternal mortality often focused on a single cause, delivered in a fragmented manner, or unsupported by evidence. Moreover, interventions utilized a facility-based approach alone where infrastructure was weak or not available. Despite having global champions for child survival, HIV/AIDS, malaria, and other health and development issues, maternal mortality had not risen to become an equal political priority.

## WHAT WAS ATTEMPTED?

On June 1, 2012, the Saving Mothers, Giving Life initiative was launched. It was a concerted response by the U.S. Government through President Barack Obama's Global Health Initiative, with its focus on women and girls and integrated responses to global health challenges. Secretary Hillary Clinton emphasized these aims by focusing on accelerating the reduction of maternal mortality in countries where the United States had a significant global health investment and presence. Saving Mothers, Giving Life was a public-private partnership that engaged the entirety of the U.S. Government—particularly the U.S. Department of State and its Office of the U.S. Global AIDS Coordinator and Health Diplomacy, the United States Agency for International Development, and the U.S. Centers for Disease Control and Prevention. SMGL leveraged the U.S. President's Emergency Plan for AIDS Relief (PEPFAR) and maternal and child health platforms, expertise, partners, and infrastructure for maximizing efficiency and impact. In addition to the U.S. Government, the founding partners included the Government of Norway, Merck, the American College of Obstetricians and Gynecologists, Project C.U.R.E., and Every Mother Counts. The Governments of Uganda and Zambia, and later, Nigeria, were also central members of the partnership at the country level.

Saving Mothers, Giving Life was a bold attempt to show that maternal mortality could be reduced significantly in developing countries. It was inspired by the progress seen by other high-level initiatives (e.g., PEPFAR, the President's Malaria Initiative, Feed the Future) that modeled how high-level political leadership, focused attention, evidence-based interventions, clear outcome data, a broad coalition, and strong monitoring and evaluation could achieve impressive results in a short time.

The initial goal of Saving Mothers, Giving Life was to support countries to reduce maternal deaths by up to 50% in targeted districts in Uganda and Zambia—particularly during the critical window during labor, delivery, and the first 24–48 hours postpartum when an estimated 2 of every 3 maternal deaths and 45% of newborn deaths occur.^1^ An audacious goal, rather than an incremental goal, was established to engender new collaborative efforts between U.S. government agencies and the partnership.

To reach these goals, the Saving Mothers, Giving Life model employed a systems approach focused at the health district level to ensure that every pregnant woman had access to clean and safe normal delivery services and, in the event of an obstetric complication, lifesaving emergency care within 2 hours. The model served to strengthen the existing public and private health networks within each district to address the “Three Delays”: delay in seeking appropriate services, delay in reaching services, and delay in receiving timely, quality care at the facility. The Saving Mothers, Giving Life approach also integrated maternal and newborn health services with HIV services (e.g., HIV counseling and testing and prevention of mother-to-child transmission services).

The global partnership sought to leverage strengths, experience, methodologies, and resources of each partner in pursuit of the Saving Mothers, Giving Life goal. The effort used an integrated approach recognizing that a health care delivery system needed to function well in real time in order to prevent maternal death. The integrated systems approach focused on the following interventions: (1) skilled attendance at birth; (2) safe facilities and hospitals for delivery; (3) supplies and provision of basic and emergency obstetric services; (4) systems for communication, referral, and transportation available 24 hours a day, 7 days a week; and (5) quality data, surveillance, and response. Over the course of the 5-year partnership, the founding partners pledged more than US$200 million in financial and in-kind resources to support the implementation of Saving Mothers, Giving Life.

## WHAT WAS ACCOMPLISHED?

The results shared in this Saving Mothers, Giving Life journal supplement show that the initiative achieved tremendous impact in Uganda and Zambia. The initiative's data-driven approach clearly resulted in improved health outcomes, including declines in maternal mortality by 44% in target facilities in Uganda and 38% in target facilities in Zambia.^2^ In addition, Uganda and Zambia both saw significant reductions in mothers dying across target districts: 44% in Uganda and 41% in Zambia.^2^ This means Saving Mothers, Giving Life did not just reach women who made it to the facility but also improved the health of mothers across the community. Further results of Saving Mothers, Giving Life include:

- Increasing the number of women delivering in health facilities in Zambia by 44% and decreasing total stillbirths in the facility by 36%.
- Increasing the number of women who are treated to prevent mother-to-child transmission by 71% in target districts in Uganda.
- Expanding home visiting programs to reach more women and newborns during the critical first few days of life and broadening training and mentoring programs on sick newborn care to ensure all providers are equipped to save lives.^2^

In addition, Saving Mothers, Giving Life offers lessons on U.S. Government interagency models and the dynamics of a public-private partnership. Most significantly, the effort relied on the dedication, expertise, and entrepreneurship of Uganda and Zambia government medical and local civic leaders accompanied by equally dedicated and talented U.S. government teams with support from the U.S. ambassadors to Uganda and Zambia. Considerable problem solving, resource gathering, and resilience in the face of unexpected administrative and logistical challenges were required.

The 11 articles presented in this supplement provide extensive detail on the model, data, impact, costs, innovations, and sustainability of the Saving Mothers, Giving Life partnership and approach:

- **Article 1**: Saving Mothers, Giving Life: It Takes a System to Save a Mother.^3^
- **Article 2:** Impact of the Saving Mothers, Giving Life Approach on Decreasing Maternal and Perinatal Deaths in Uganda and Zambia.^4^
- **Article 3:** Addressing the First Delay in Saving Mothers, Giving Life Districts in Uganda and Zambia: Approaches and Results for Increasing Demand for Facility Delivery Services.^5^
- **Article 4:** Addressing the Second Delay in Saving Mothers, Giving Life Districts in Uganda and Zambia: Reaching Appropriate Maternal Care in a Timely Manner.^6^
- **Article 5:** Addressing the Third Delay in Saving Mothers, Giving Life Districts in Uganda and Zambia: Ensuring Adequate and Appropriate Facility-Based Maternal and Perinatal Health Care.^7^
- **Article 6:** The Costs and Cost-Effectiveness of a District Strengthening Strategy to Mitigate the 3 Delays to Quality Maternal Health Care: Results From Uganda and Zambia.^8^
- **Article 7:** Saving Lives Together: A Qualitative Evaluation of the Saving Mothers, Giving Life Public-Private Partnership.^9^
- **Article 8:** Community Perceptions of a 3-Delays Model Intervention: A Qualitative Evaluation of Saving Mothers, Giving Life in Zambia.^10^
- **Article 9:** Did Saving Mothers, Giving Life Expand Timely Access to Lifesaving Care in Uganda? A Spatial District-Level Analysis of Travel Time to Emergency Obstetric and Newborn Care.^11^
- **Article 10:** Saving Mothers, Giving Life Approach for Strengthening Health Systems to Reduce Maternal and Newborn Deaths in 7 Scale-up Districts in Northern Uganda.^12^
- **Article 11:** Sustainability and Scale of the Saving Mothers, Giving Life Approach in Uganda and Zambia.^13^

## CONCLUSION

In conclusion, the Saving Mothers, Giving Life partnership and approach resulted in a focused, systematic, district-level program driven by data and results-orientation for reducing maternal mortality. The approach and subsequent impacts underscore the importance of investing in health systems to not only sustainably save mothers and newborns but also make systems more resilient so they can address other emerging health issues requiring an integrated approach, such as cardiovascular disease, diabetes, and motor vehicle crashes.

Although the 5-year partnership is coming to an end, key elements of the effort are still being sustained in country programming. As we look into the future, the journey remains long. We must sustain the momentum and work together as a global community to maintain the focus on reducing maternal mortality in a data-driven and focused manner. As the African proverb states, “If you want to go fast, go alone. If you want to go far, go with others.” The long list of those involved in the Saving Mothers, Giving Life Working Group, in the acknowledgments below, confirms that the initiative's goal was to mobilize many to go far. Ending preventable maternal and newborn deaths will require that we continue on this journey together until these tragic deaths are history.

## Notes

**Peer Reviewed**

**Funding:** Saving Mothers, Giving Life implementation was primarily funded by the Office of the Global AIDS Coordinator, the U.S. Agency for International Development (USAID), Washington, DC, the Centers for Disease Control and Prevention (CDC), Atlanta, Georgia (Cooperative agreements GPS003057 and GPS002918), Merck for Mothers, and Every Mother Counts. The funding agencies had no influence or control over the content of this article.
